# Eviprostat has an identical effect compared to pollen extract (Cernilton) in patients with chronic prostatitis/chronic pelvic pain syndrome: a randomized, prospective study

**DOI:** 10.1186/s12894-015-0115-5

**Published:** 2015-12-07

**Authors:** Hiromichi Iwamura, Takuya Koie, Osamu Soma, Teppei Matsumoto, Atsushi Imai, Shingo Hatakeyama, Takahiro Yoneyama, Yasuhiro Hashimoto, Chikara Ohyama

**Affiliations:** Department of Urology, Hirosaki University Graduate School of Medicine, 5 Zaifucho, Hirosaki, Aomori 036-8562 Japan

**Keywords:** Chronic prostatitis/chronic pelvic pain syndrome, Eviprostat, Pollen extract

## Abstract

**Background:**

Previously reported results of a prospective, randomized placebo-controlled study showed that the pollen extract (Cernilton) significantly improved total symptoms, pain, and quality of life in patients with inflammatory prostatitis/chronic pelvic pain syndrome (CP/CPPS) without severe side effects. A phytotherapeutic agent, Eviprostat, is reportedly effective in a rat model of nonbacterial prostatitis. The aim of the present study was to compare the efficacy and safety of Eviprostat to that of the pollen extract in the management of CP/CPPS.

**Methods:**

The patients with category III CP/CPPS were randomized to receive either oral capsules of Eviprostat (two capsules, q 8 h) or the pollen extract (two capsules, q 8 h) for 8 weeks. The primary endpoint of the study was symptomatic improvement in the NIH Chronic Prostatitis Symptom Index (NIH-CPSI). Participants were evaluated using the NIH-CPSI and the International Prostate Symptom Score (IPSS) at baseline and after 4 and 8 weeks.

**Results:**

In the intention-to-treat analysis, 100 men were randomly allocated to Eviprostat (*n* = 50) or the pollen extract (*n* = 50). Response (defined as a decrease in the NIH-CPSI total score by at least 25 %) in the Eviprostat group and the pollen extract group was 88.2 and 78.1 %, respectively. There was no significant difference in the total, pain, urinary, and quality of life (QOL) scores of the NIH-CPSI between the two groups at 8 weeks. This was also the case with the total, voiding, and storage symptoms of the IPSS. There were no severe adverse events observed in any patients in this study.

**Conclusion:**

Both the pollen extract and Eviprostat significantly reduced the symptoms of category III CP/CPPS without any adverse events. Eviprostat may have an identical effect on category III CP/CPPS compared the pollen extract.

**Trial registration:**

The study was registered with the University Hospital Medical Information Network Clinical Trials Registry in Japan (UMIN000019618); registration date: 3 November 2015.

## Background

Prostatitis is a relatively common urological disease that occurs in adult men [1]. The U.S. National Institutes of Health (NIH) Advisory Committees divided prostatitis into four categories [2, 3]. Of these, the incidence of category III disease, chronic prostatitis/chronic pelvic pain syndrome (CP/CPPS) is believed to be very high [1]. Category III prostatitis is subdivided into the inflammatory type (IIIA; similar to nonbacterial CP) and non-inflammatory type (IIIB; similar to prostatodynia) based on the presence (IIIA) or absence (IIIB) of leukocytes in prostatic secretions or seminal plasma [2, 3].

While the cause of CP/CPPS is presently unknown, it is a disease that has many clinical issues because it is often resistant to various treatments [4–6]. To date, CP/CPPS has been treated using alpha-blockers, antibacterial agents, anti-inflammatory agents, and phytotherapeutic agents with varying outcomes [4–12]. Phytotherapeutic agents that have been used include pollen extract, quercetin, and saw palmetto. Several years ago, Wagenlehner FM et al. announced the results of a prospective, randomized placebo-controlled study, which indicated that the pollen extract (Cernilton) significantly improved the total symptoms, pain, and quality of life in patients with inflammatory prostatitis/chronic pelvic pain syndrome (CP/CPPS) without any severe adverse effects [6].

Eviprostat is a phytotherapeutic agent widely used in the treatment of prostatic hypertrophy and has been used in Japan and Germany for more than 40 years [13–15]. Eviprostat consists of five components: four are extracted from the umbellate wintergreen Chimaphila umbellata, the aspen Populus tremula, the small pasque flower Pulsatilla pratensis, and the field horsetail Equisetum arvense, and the fifth is germ oil from wheat (Tritium aestivum) [13–15].

Oka et al. administered Eviprostat treatment in a rat model of nonbacterial prostatitis and reported that oxidative stress and proinflammatory cytokines in the enlarged prostate were considerably suppressed, and that Eviprostat may be useful in the clinical treatment of CP/CPPS [13–15]. Here we conducted a randomized prospective study to determine the effectiveness and safety of Eviprostat to treat CP/CPPS in comparison with pollen extract.

## Methods

### Study design

This double-blind, prospective, randomized and multicentre clinical phase 3 study was conducted in 8 Japan urologic centers to ascertain the safety and efficacy of 8-weeks Eviprostat in men diagnosed with inflammatory CP/CPPS.

The design of the study was in accordance with the guidelines for clinical trials in CP/CPPS described by the NIH Chronic Prostatitis Collaborative Research Network [16].

Inclusion criteria were [1] men between 20 and 80 year of age with symptoms of pelvic pain for 3 months or more before study [2]. Patients with a total National Institutes of Health Chronic Prostatitis Symptom Index (NIH-CPSI) score ≥15 point [3]. Patients diagnosed with NIH category IIIA and IIIB using the PPMT (pre- and post-massage test) . Category IIIA refers to the presence of white blood cells (WBC) after a prostate massage urine specimen (VB3) (WBC in VB3 > 10/hps). Category IIIB refers to patients with pelvic pain with no evidence of inflammation on VB3.

Exclusion criteria were [1] documented urinary tract infection (midstream urine culture with at least 100,000 colony-forming units per milliliter), [2] history of urethritis, epididymitis or sexually transmitted disease (STD) [3] history of prostate surgery [4] history of urogenital cancer [5] treatment with phytotherapeutic agents, a-blocker agents, or antimicrobials. [6] residual urine volume >50 ml resulting from bladder outlet obstruction (BOO).

The study protocol was approved by the ethical committee of Hirosaki University School of Medicine, Aomori, Japan. Written informed consent was obtained from all patients to participation in this study. This study was registered with the Hirosaki University Hospital Clinical Trials Registry in Japan (2009-013) on 24 May 2009 and was registered with the University Hospital Medical Information Network Clinical Trials Registry in Japan (UMIN000019618) on 3 November 2015.

### Study procedure

We included in our study patients with urinary symptoms who met our inclusion criteria from among patients who had been diagnosed with clinically chronic prostatitis in medical interviews. The significance, objectives, and methods of this clinical study were fully explained to the patients, and their voluntary written informed consent was obtained. The patients’ subjective symptoms were evaluated using NIH-CPSI (Japanese version) and International Prostate Symptom Score (IPSS) (Japanese version) [17, 18].

We checked patients 1 week after initiating drug therapy to ascertain whether they met the inclusion criteria. Patients were then allocated to receive either Eviprostat [two capsules q8h, with the active substance consisting of the umbellate wintergreen Chimaphila umbellate extract 0.5 mg, the aspen Populus tremula extract 0.5 mg, the small pasque flower Pulsatilla pratensis extract 0.5 mg, the field horsetail Equisetum arvense extract 1.5 mg and germ oil from wheat (Tritium aestivum) 15.0 mg.] or pollen extract (two capsules q8h, with the active substance consisting of 60 mg Cernitin T60 and 3 mg Cernitin GBX) The allocation manager randomly determined which of the 2 drugs would be administered to each patient. Cards detailing the drug to be used were sealed in numbered envelopes and distributed to patients from the smallest number to the largest. The drug to be used was decided on the basis of the card.

### Statistical analysis

We used the SPSS 21.0 software package (SPSS, Chicago, IL) for statistical analyses. Intergroup differences were analyzed by the Student’s *t*-test. Intragroup differences were analyzed by a paired *t*-test. A value of *P* < 0.05 was considered statistically significant.

## Results

**Fig. 1:**
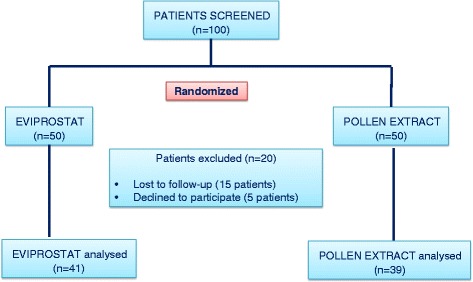
Flow chart of this study

We randomized 100 patients diagnosed Category III A/ III B prostatitis. 80 patients completed 12 weeks of follow-up and had primary and secondary outcomes ascertained. Flow chart of this study was presented in Fig. 1. In Eviprostat group, 7 patients were lost to follow-up and 2 patients declined to participate the study. In pollen extract group, 8 patients were lost to follow up and 3 patients declined to participate the study.

In Eviprostat group, there were 26 category IIIA patients and 15 category IIIB patients. In pollen extract group, there were 20 category IIIA patients and 19 category IIIB patients. There were no differences from baseline in the number of leukocytes in the prostatic secretion between the two groups.

The baseline characteristics of each study group are presented in Table 1. In the Quality of Life (QOL) domain of NIH-CPSI, there were significant differences between two groups. (*p* = 0.014) Except for QOL domain, there were no significant differences between the two groups at the start of this study.

**Table 1:** Patients background

	Eviprostat	Pollen extract	p value
Number	41	39	n.s.
Age	50.1 ± 13.7	53.0 ± 14.6	n.s.
CategoryIIIA/IIIB	26/15	20/19	n.s.
Duration of current symptoms (months)	8.2 ± 10.6	9.5 ± 11.2	n.s.
IPSS	10.8 ± 7.5	11.6 ± 7.3	n.s.
NIH-CPSI			
Total score	22.3 ± 4.7	20.3 ± 5.8	n.s.
Pain domain	9.4 ± 4.2	9.2 ± 4.0	n.s.
Urinary domain	4.6 ± 2.8	3.8 ± 2.7	n.s.
QoL domain	8.3 ± 1.6	7.3 ± 2.0	0.014

**Fig. 2:**
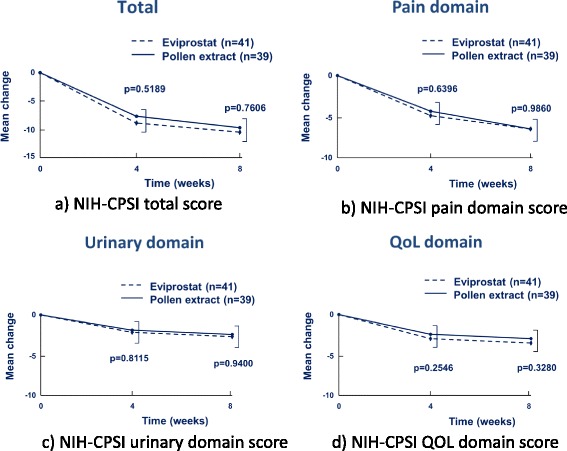
Mean change from baseline in the NIH-CPSI total score and in the sub-score after 4 and 8 week of treatment with Cernilton group or Eviprostat group. **a** NIH-CPSI total score. **b** NIH-CPSI pain domain score. **c** NIH-CPSI urinary domain score. **d** NIH-CPSI QOL domain score

**Fig. 3:**
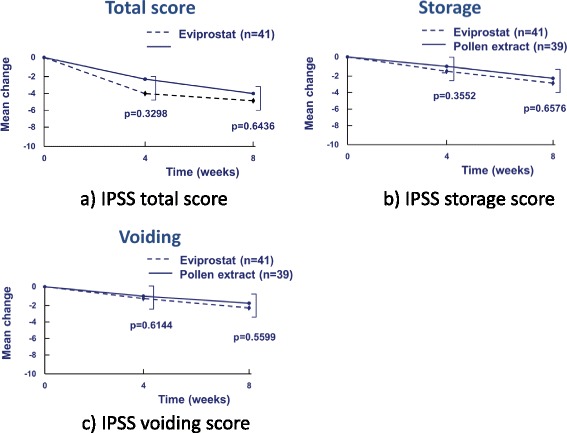
Mean change from baseline in the IPSS total score and in the sub-score after 4 and 8 week of treatment with Cernilton group or Eviprostat group. **a** IPSS total score. **b** IPSS storage score. **c** IPSS voiding score

Response (defined as a decrease in the NIH-CPSI total score by at least 25 %) in the Eviprostat group and the pollen extract group at 4 week was 68.3 and 61.5 %, respectively. Response in the Eviprostat group and the pollen extract group was 88.2 and 78.1 %, respectively. There were no severe adverse events observed in any patients in this study (Table 2). There was no significant difference in the total, pain, urinary, and the QOL scores of the NIH-CPSI between the two groups at 4 weeks and 8 weeks (Fig. 2). There were no significant differences about the total, voiding, and storage symptoms of the IPSS between two groups (Fig. 3). There were no severe adverse events observed in any patients in this study.

**Table 2:** 25% response rates for NIH-CPSI

	Eviprostat		Pollen extract	
	4 weeks	8 weeks	4 weeks	8 weeks
Total variation	-8.9	-11.1	-7.8	-10.5
Adverse event (%)	1.7	2.3	2.3	4.7
25 % response rates (%)	68.3	88.2	61.5	78.1

## Discussion

Antibiotics administration is the standard treatment for chronic bacterial prostatitis [19], however, the standard treatment for CP/CPPS has not yet been established [20]. To date, various treatments for CP/CPPS have been reported, including α-blockers, antibiotics, anti-inflammatory agents, phytotherapeutics, and various other modalities [4–12]. However it is believed that these treatments have little effect on major symptoms, such as pain and urinary disturbance, experienced in CP/CPPS that reduce the QOL [21].

In general, patients with CP/CPPS undergo long-term treatment, and therefore, phytotherapeutics such as pollen extract, quercetin, Saw palmetto, or terpenes may be useful because they have few side effects [5]. However, there is no scientific evidence supporting these agents, and only few prospective controlled clinical trials have been conducted.

Since a long time, Cernilton has been used for the treatment of prostatitis [6]. Wagenlehner et al. conducted a prospective, randomized, double-blind, placebo-controlled study to study the effect of Cernilton in patients with CP/CPPS (NIH IIIA). They reported that compared with a placebo, Certilton improved total symptom, pain, and QOL without any side effects [6].

Eviprostat is a phytotherapeutic agent commonly used to treat prostatic hypertrophy in Japan [13–15]. An experiment using nonbacterial prostatitis model suggested that Evoprostat is potentially effective for the treatment of CP/CPPS. Oka et al previously reported that by using a model of non-bacterial prostatitis (NBP) induced in castrated aging rats by the injection of 17b-estradiol, they showed that the increased production of oxidativestress marker malondialdehyde (MDA) and the proinflammatory cytokines TNF-a, IL-6, and IL-8 in prostate tissue homogenates from NBP rats. Eviprostat treatment significantly suppressed oxidative stress and proinflammatory cytokines in the NBP rats [13]. Sugimoto et al reported that chemokines, including CCL2/MCP-1 and CXCL1/CINC-1, were elevated in the prostate and urine of NBP rats, and Eviprostat potently suppressed the increases in CCL2/MCP-1 and CXCL1/CINC-1 [14].

The aim of the present study was to compare the efficacy and safety of Eviprostat to that of the pollen extract in the management of CP/CPPS.

In the intention-to-treat analysis, 100 Category III CP/CPPS patients were randomly allocated to Eviprostat (*n* = 50) or the pollen extract (*n* = 50). Response (defined as a decrease in the NIH-CPSI total score by at least 25 %) in the Eviprostat group and the pollen extract group was 88.2 and 78.1 %, respectively. There was no significant difference in the total, pain, urinary, and QOL scores of the NIH-CPSI between the two groups at 8 weeks.

This study has several limitations. Study samples were very small, it is necessary to examine the therapeutic effects of Eviprostat with a placebo control and this study was conducted in only Japanese populations.

In the present study, we conducted a prospective, randomized trial to compare the therapeutic effects of Eviprostat and Certilton, the standard treatment for CP/CPPS in Japan, and found that both agents improved CP/CPPS without any side-effects. We believe that Eviprostat is a very promising phytotherapeutic agent for the treatment of CP/CPPS in the future.

## Conclusion

Both the pollen extract and Eviprostat significantly reduced the symptoms of category III CP/CPPS without any adverse events. Eviprostat may have an identical effect on category III CP/CPPS compared the pollen extract.

## Abbreviations

CP: chronic prostatitis
CPPS: chronic pelvic pain syndrome
hps: high-power field
IPSS: International Prostate Symptom Index
MDA: malondialdehyde
NBP: non-bacterial prostatitis
NIH: National Institutes of Health
NIH-CPSI: NIH-Chronic Prostatitis Symptom Index
PPMT: pre and post massage test
QOL: quality of life
STD: sexually transmitted disease
VB3: prostate massage urine specimen
WBC: white blood cells

## References

[1] Anothaisintawee T, Attia J, Nickel JC, Thammakraisorn S, Numthavaj P, McEvoy M (2011). Management of chronic prostatitis/chronic pelvic pain syndrome: a systematic review and network meta-analysis. JAMA.

[2] Krieger JN, Nyberg L, Nickel JC (1999). NIH consensus definition and classification of prostatitis. JAMA.

[3] Fu W, Zhou Z, Liu S, Li Q, Yao J, Li W (2014). The effect of chronic prostatitis/chronic pelvic pain syndrome (CP/CPPS) on semen parameters in human males: a systematic review and meta-analysis. PLoS One.

[4] Nickel JC, Krieger JN, McNaughton-Collins M, Anderson RU, Pontari M, Shoskes DA (2008). Alfuzosin and symptoms of chronic prostatitis-chronic pelvic pain syndrome. N Engl J Med.

[5] Nickel JC (2008). Treatment of chronic prostatitis/chronic pelvic pain syndrome. Int J Antimicrob Agents.

[6] Wagenlehner FM, Schneider H, Ludwig M, Schnitker J, Brahler E, Weidner W (2009). A pollen extract (Cernilton) in patients with inflammatory chronic prostatitis-chronic pelvic pain syndrome: a multicentre, randomised, prospective, double-blind, placebo-controlled phase 3 study. Eur Urol.

[7] Thakkinstian A, Attia J, Anothaisintawee T, Nickel JC (2012). alpha-blockers, antibiotics and anti-inflammatories have a role in the management of chronic prostatitis/chronic pelvic pain syndrome. BJU Int.

[8] Nickel JC, Downey J, Clark J, Casey RW, Pommerville PJ, Barkin J (2003). Levofloxacin for chronic prostatitis/chronic pelvic pain syndrome in men: a randomized placebo-controlled multicenter trial. Urology.

[9] Bates SM, Hill VA, Anderson JB, Chapple CR, Spence R, Ryan C (2007). A prospective, randomized, double-blind trial to evaluate the role of a short reducing course of oral corticosteroid therapy in the treatment of chronic prostatitis/chronic pelvic pain syndrome. BJU Int.

[10] Jeong CW, Lim DJ, Son H, Lee SE, Jeong H (2008). Treatment for chronic prostatitis/chronic pelvic pain syndrome: levofloxacin, doxazosin and their combination. Urol Int.

[11] Nickel JC, Narayan P, McKay J, Doyle C (2004). Treatment of chronic prostatitis/chronic pelvic pain syndrome with tamsulosin: a randomized double blind trial. J Urol.

[12] Nickel JC, Pontari M, Moon T, Gittelman M, Malek G, Farrington J (2003). A randomized, placebo controlled, multicenter study to evaluate the safety and efficacy of rofecoxib in the treatment of chronic nonbacterial prostatitis. J Urol.

[13] Oka M, Ueda M, Oyama T, Kyotani J, Tanaka M (2009). Effect of the phytotherapeutic agent Eviprostat on 17beta-estradiol-induced nonbacterial inflammation in the rat prostate. Prostate.

[14] Sugimoto M, Oka M, Tsunemori H, Yamashita M, Kakehi Y (2011). Effect of a phytotherapeutic agent, Eviprostat(R), on prostatic and urinary cytokines/chemokines in a rat model of nonbacterial prostatitis. Prostate.

[15] Tsunemori H, Sugimoto M, Xia Z, Taoka R, Oka M, Kakehi Y (2011). Effect of the phytotherapeutic agent Eviprostat on inflammatory changes and cytokine production in a rat model of nonbacterial prostatitis. Urology.

[16] Propert KJ, Alexander RB, Nickel JC, Kusek JW, Litwin MS, Landis JR (2002). Design of a multicenter randomized clinical trial for chronic prostatitis/chronic pelvic pain syndrome. Urology.

[17] Monden K, Tsugawa M, Ninomiya Y, Ando E, Kumon H (2002). A Japanese version of the National Institutes of Health Chronic Prostatitis Symptom Index (NIH-CPSI, Okayama version) and the clinical evaluation of cernitin pollen extract for chronic non-bacterial prostatitis. Nihon Hinyokika Gakkai Zasshi.

[18] Homma Y, Tsukamoto T, Yasuda K, Ozono S, Yoshida M, Shinji M (2002). Linguistic validation of Japanese version of International Prostate Symptom Score and BPH impact index. Nihon Hinyokika Gakkai Zasshi.

[19] Bjerklund Johansen TE, Gruneberg RN, Guibert J, Hofstetter A, Lobel B, Naber KG (1998). The role of antibiotics in the treatment of chronic prostatitis: a consensus statement. Eur Urol.

[20] Tugcu V, Tasci AI, Fazlioglu A, Gurbuz G, Ozbek E, Sahin S (2007). A placebo-controlled comparison of the efficiency of triple- and monotherapy in category III B chronic pelvic pain syndrome (CPPS). Eur Urol.

[21] Nickel JC (2008). Role of alpha1-blockers in chronic prostatitis syndromes. BJU Int.

